# Unveiling evolving surgical landscape amidst the pandemic: a comprehensive analysis

**DOI:** 10.1016/j.bjorl.2023.101329

**Published:** 2023-09-15

**Authors:** Fábio Lobato de Campos Oliveira, Renato Assis Machado, Rubens Gonçalves Teixeira

**Affiliations:** a Faculdade de Odontologia São Leopoldo Mandic, Departamento de Cirurgia Oral e Maxilofacial, Campinas, SP, Brazil; b Universidade de Campinas (UNICAMP), Faculdade de Odontologia de Piracicaba (FOP) e Programa de Pós-Graduação em Biologia Oral, Departamento de Diagnóstico Oral, Piracicaba, SP, Brazil

Dear Editor,

The global healthcare landscape underwent significant transformations in response to the COVID-19 pandemic, marked by resource constraints and the deferment of elective surgeries to alleviate strain on healthcare systems. The field of Oral and Maxillofacial Surgery also experienced these shifts, prompting the need to comprehend patient profiles to inform preventive measures and planning strategies. A retrospective examination, approved by the research ethics committee under Protocol nº 5,875,699, delved into medical records of patients undergoing facial trauma surgeries at a prominent Minas Gerais hospital between January 2018 and December 2021. The study scrutinized surgical procedures and their underlying determinants across two distinct epochs: the pre-pandemic and pandemic eras.

Interestingly, the pandemic triggered a slight reduction in the average age of patients undergoing facial trauma surgeries. Male patients remained dominant in both periods, albeit with a minor decline during the pandemic. Surprisingly, the pandemic prompted an increased reliance on the public healthcare system (Unified Health System), juxtaposed with a notable decline in health plan coverage (−23.1%) (Table 1).

**Table 1:** Characteristics of the patients included in the study.

	2018	2019	2018‒2019	2020	2021	2020‒2021	Difference between Pre- and COVID-19 pandemic period
	n (%)	n (%)	n (%)	n (%)	n (%)	n (%)	
Sex							
Male	146 (86.9)	142 (80.7)	288 (83.7)	144 (82.7)	138 (84.1%)	282 (83.4)	(−) 2.1%
Female	22 (13.1)	34 (19.3)	56 (16.3)	30 (17.3)	26 (15.9)	56 (16.6)	0.0%
Age	43.1 (±18.6; 7‒86)	40.9 (±15.9; 12‒93)	42.0 (±17.2; 7‒93)	38.9 (±16.7; 11‒84)	39.1 (±15.5; 10‒80)	39.0 (±16.1; 10‒84)	(−) 7.1%
Health insurance							
Public	151 (89.9)	154 (87.5)	305 (88.7)	161 (92.5)	147 (89.6)	308 (91.1)	(+) 1.0%
Private	17 (10.1)	22 (12.5)	39 (11.3)	13 (7.5)	17 (10.4)	30 (8.9)	(−) 23.1%

Etiological factors were systematically categorized into fourteen groups. Notable reductions emerged in incidents involving animal-related accidents (−25.0%), falls from heights (−17.6%), firearm injuries (−53.8%), physical assaults (−24.6%), vehicular collisions (−50.0%), and sports-related accidents (−62.5%) during the pandemic. In contrast, traffic-related accidents, such as car (+24.0%), cycling (+25.7%), and motorcycle accidents (+18.3%), escalated. Likewise, falls from one's height surged (+29.4%) (Table 2).

**Table 2:** Difference between the types of accidents in the pre- and during the COVID-19 pandemic.

	2018	2019	2018‒2019	2020	2021	2020‒2021	Difference between Pre- and COVID-19 pandemic period
	n (%)	n (%)	n (%)	n (%)	n (%)	n (%)	
Animals accident	6 (3.6)	10 (5.7)	16 (4.7)	5 (2.9)	7 (4.3)	12 (3.6)	(−) 25.0%
Car accident	15 (8.9)	23 (13.1)	38 (11.0)	22 (12.6)	28 (17.1)	50 (14.8)	(+) 24.0%
Cycling accident	18 (10.7)	8 (4.5)	26 (7.6)	19 (10.9)	16 (9.8)	35 (10.4)	(+) 25.7%
Domestic accident	4 (2.4)	‒	4 (1.2)	1 (0.6)	3 (1.8)	4 (1.2)	0.0%
Fall from height	10 (5.9)	7 (4.0)	17 (4.9)	4 (2.3)	10 (6.1)	14 (4.1)	(−) 17.6%
Fall from own height	16 (9.5)	8 (4.5)	24 (7.0)	21 (12.1)	13 (7.9)	34 (10.1)	(+) 29.4%
Fracture after tooth extraction	‒	‒	‒	1 (0.6)	1 (0.6)	2 (0.6)	‒
Injuries by firearms	7 (4.2)	6 (3.4)	13 (3.8)	2 (1.1)	4 (2.4)	6 (1.8)	(−) 53.8%
Motorcycle accident	27 (16.1)	22 (12.5)	49 (14.2)	28 (16.1)	32 (19.5)	60 (17.8)	(+) 18.3%
Physical aggression	27 (16.1)	30 (17.0)	57 (16.6)	17 (9.8)	26 (15.8)	43 (12.7)	(−) 24.6%
Running over	3 (1.8)	9 (5.1)	12 (3.5)	2 (1.1)	4 (2.4)	6 (1.8)	(−) 50.0%
Sport accident	5 (2.9)	3 (1.7)	8 (2.3)	2 (1.1)	1 (0.6)	3 (0.9)	(−) 62.5%
Suicide	1 (0.6)	‒	1 (0.3)	‒	‒	‒	‒
Work accident	4 (2.4)	5 (2.8)	9 (2.6)	5 (2.9)	4 (2.4)	9 (2.7)	0.0%
No information	25 (14.9)	45 (25.7)	70 (20.0)	45 (25.9)	15 (9.1)	60 (17.8)	‒
Total	168	176	344	174	164	338	(−) 1.7%

Fracture type distribution remained largely consistent, except for a significant rise in naso-orbito-ethmoidal complex fractures (+90.0%; *p*-value = 0.006). Facial polytrauma (+27.7%), foreign body removal (+100.0%), multiple mandibular fractures (+9.8%), and simple mandibular fractures (+5.0%) experienced an upward trajectory. Conversely, certain injuries like blunt stump injury (−66.7%), comminuted mandibular fractures (−100.0%), frontal bone fractures (−50.0%), Le Fort II maxillary fractures (−66.7%), nasal fractures (−24.4%), and orbital floor fractures (−66.7%) exhibited moderate declines (Table 3). Furthermore, an increase in the use of plates and screws for fixation was evident, while surgeries not requiring orthotic and prosthetic materials declined notably (−14.9%) (Table 4). These findings illuminate the fluid shifts in surgical procedures and their driving factors during the pandemic.

**Table 3:** Difference between the types of fracture in the pre- and during the COVID-19 pandemic.

	2018	2019	2018‒2019	2020	2021	2020‒2021	Difference between Pre- and COVID-19 pandemic period
	n (%)	n (%)	n (%)	n (%)	n (%)	n (%)	
Blunt Stump Injury	3 (1.8)	‒	3 (0.9)	‒	1 (0.6)	1 (0.3)	(−) 66.7%
Comminutive fracture of the mandible	3 (1.8)	2 (1.1)	5 (1.5)	‒	‒	‒	(−) 100.0%^a^
Complex zigomatic fracture	45 (26.8)	56 (31.8)	101 (29.4)	51 (29.3)	50 (30.5)	101 (29.9)	0.0%
Dental alveolus fracture	1 (0.6)	6 (3.4)	7 (2.0)	4 (2.3)	3 (1.8)	7 (2.1)	0.0%
Facial polytrauma	18 (10.7)	16 (9.1)	34 (9.9)	25 (14.4)	22 (13.4)	47 (13.9)	(+) 27.7%
Foreign body removal	‒	‒	‒	1 (1.1)	‒	1 (0.3)	(+) 100.0
Fracture of frontal	2 (1.2)	4 (2.3)	6 (1.8)	2 (1.1)	1 (0.6)	3 (0.9)	(−) 50.0%
Lanelong maxillary fracture	‒	1 (0.6)	1 (0.3)	‒	‒	‒	(−) 100.0
Maxillary fracture Le Fort I	1 (0.6)	1 (0.6)	2 (0.6)	‒	2 (1.2)	2 (0.6)	0.0%
Maxillary fracture Le Fort II	6 (3.6)	3 (1.7)	9 (2.6)	1 (0.6)	2 (1.2)	3 (0.9)	(−) 66.7%
Maxillary fracture Le Fort III	1 (0.6)	3 (1.7)	4 (1.2)	2 (1.1)	2 (1.2)	4 (1.2)	0.0%
Multiple fracture of mandible	23 (13.7)	23 (13.1)	46 (13.4)	25 (14.4)	26 (15.9)	51 (15.1)	(+) 9.8%
Nasal fracture	25 (14.9)	20 (11.4)	45 (13.1)	21 (12.1)	13 (7.9)	34 (10.1)	(−) 24.4%
Naso Orbital Etthmoidal fracture	1 (0.6)	‒	1 (0.3)	2 (1.1)	8 (4.9)	10 (3.0)	(+) 90.0%^b^
Orbital floor fracture	4 (2.4)	5 (2.8)	9 (2.6)	3 (1.7)	‒	3 (0.9)	(−) 66.7%
Simple fracture of the mandible	28 (16.7)	29 (16.5)	57 (16.6)	29 (16.7)	31 (18.9)	60 (17.8)	(+) 5.0%
Zygomatic arch fracture	4 (2.4)	7 (4.0)	11 (3.2)	8 (4.6)	3 (1.8)	11 (3.3)	0.0%
No information	3 (1.8)	‒	3 (0.9)	‒	‒	‒	(−) 100.0
Total	168	176	344	174	164	338	(−) 1.7%

a *p*-value < 0.02.

b *p*-value < 0.006.

**Table 4:** Difference between the need to use board Orthosis, Prothesis and Special Materials in the pre- and during the COVID-19 pandemic.

	2018	2019	2018‒2019	2020	2021	2020‒2021	Difference between Pre- and COVID-19 pandemic period
	n (%)	n (%)	n (%)	n (%)	n (%)	n (%)	
Yes	130 (77.4)	140 (79.5)	270 (78.5)	131 (75.3)	144 (87.8)	275 (81.4)	(+) 1.8%
No	38 (22.6)	36 (20.5)	74 (21.5)	43 (24.7)	20 (12.2)	63 (18.6)	(−) 14.9%
Total	168	176	344	174	164	338	(−) 1.7%

Comparison with study of Pagotto et al.^1^ highlighted that emergency trauma services were relatively less affected by the pandemic, although elective procedures' suspension and pandemic-induced psychological effects on surgeons were prevalent across specialties. This underscores the necessity for comprehensive strategies to address both immediate and long-term impacts of the pandemic on surgical training and patient care.

Despite limitations like its retrospective nature and focus on a single hospital, our study contributes significant insights into the intricate interplay between the COVID-19 pandemic, patient demographics, causal factors, and surgical practices within Oral and Maxillofacial Surgery. Amidst the ongoing pandemic and future healthcare challenges, a deeper understanding of these dynamics remains pivotal for informed adaptations, ensuring the continuity of high-quality patient care.

## Funding

There was no source of funding for the research.

## Conflicts of interest

The authors declare no conflicts of interest.
